# New Appliances and Things Medical

**Published:** 1902-04-19

**Authors:** 


### NEW APPLIANCES AND THINGS MEDICAL.

**[We shall be glad to receive, at our Office, 28 & 29 Southampton Street, Strand, London, W.C., from the manufacturers, specimens of all new preparations and appliances which may be brought out from time to time.]**

### MACKENZIE DAVIDSON'S NEW PATENT MOTOR MERCURY BREAK.

(Harry W. Cox, Limited, 9, 10 and 11 Cursitor Street, Chancery Lane, E.C.)

This motor mercury break for use with the X-ray apparatus is a great improvement upon many of the breaks in common use. As is well known in the ordinary break used with the earlier forms of coil not only were there many difficulties with the mechanical arrangements of the contact but, and this was even more vexatious, the slightest variation in the resistance was apt to alter both the length and character of the spark. Many contrivances have been devised with the object of running the break apparatus entirely independently of the coil and certainly this is the best of those which we have come across. The "make and break" is effected by a rotating blade dipping into and rising out of a mercury bath and actuated by a small motor which is run by an entirely independent battery. When rotating the impact upon the mercury is sufficiently firm to ensure an absolutely sudden and complete metallic circuit, while the break of the circuit on the arm leaving the mercury is of necessity equally complete. Thus, whatever strength of current is being used for the coil and whatever the length of shock which is being employed, we have the power of maintaining a steady speed and of ensuring all the time a complete break. Now it is on the suddenness and completeness of the interruption that the brilliancy of the spark in large degree depends, and herein lies the great beauty of this machine. The speed of the motor is regulated by a small resistance and there seems no reason why, if one recorded the musical note of the "hum," one should not be able at any time to reproduce exactly the same conditions at any future sitting. There are many other contrivances which make it a very handy working machine. The apparatus is made by Harry W. Cox, Limited, who, we understand, have supplied X-ray apparatus to several hospitals.

